# Assembly and Architecture of the EBV B Cell Entry Triggering Complex

**DOI:** 10.1371/journal.ppat.1004309

**Published:** 2014-08-21

**Authors:** Karthik Sathiyamoorthy, Jiansen Jiang, Yao Xiong Hu, Cynthia L. Rowe, Britta S. Möhl, Jia Chen, Wei Jiang, Elizabeth D. Mellins, Richard Longnecker, Z. Hong Zhou, Theodore S. Jardetzky

**Affiliations:** 1 Department of Structural Biology, Stanford University School of Medicine, Stanford, California, United States of America; 2 Department of Microbiology, Immunology & Molecular Genetics, University of California Los Angeles, Los Angeles, California, United States of America; 3 California NanoSystems Institute, University of California Los Angeles, Los Angeles, California, United States of America; 4 Department of Microbiology and Immunology, Feinberg School of Medicine, Northwestern University, Chicago, Illinois, United States of America; 5 Department of Pediatrics, Program in Immunology, Stanford University School of Medicine, Stanford, California, United States of America; Louisiana State University Health Sciences Center, United States of America

## Abstract

Epstein-Barr Virus (EBV) is an enveloped double-stranded DNA virus of the *gammaherpesvirinae* sub-family that predominantly infects humans through epithelial cells and B cells. Three EBV glycoproteins, gH, gL and gp42, form a complex that targets EBV infection of B cells. Human leukocyte antigen (HLA) class II molecules expressed on B cells serve as the receptor for gp42, triggering membrane fusion and virus entry. The mechanistic role of gHgL in herpesvirus entry has been largely unresolved, but it is thought to regulate the activation of the virally-encoded gB protein, which acts as the primary fusogen. Here we study the assembly and function of the reconstituted B cell entry complex comprised of gHgL, gp42 and HLA class II. The structure from negative-stain electron microscopy provides a detailed snapshot of an intermediate state in EBV entry and highlights the potential for the triggering complex to bring the two membrane bilayers into proximity. Furthermore, gHgL interacts with a previously identified, functionally important hydrophobic pocket on gp42, defining the overall architecture of the complex and playing a critical role in membrane fusion activation. We propose a macroscopic model of the initiating events in EBV B cell fusion centered on the formation of the triggering complex in the context of both viral and host membranes. This model suggests how the triggering complex may bridge the two membrane bilayers, orienting critical regions of the N- and C- terminal ends of gHgL to promote the activation of gB and efficient membrane fusion.

## Author Summary

The various steps by which lipid-enveloped viruses enter or ‘fuse’ with a host cell require the coordination of receptor recognition, viral protein activation and large protein conformational changes that can drive membrane bilayer fusion. Here we report biochemical, structural and functional experiments on the protein complex that triggers activation of the EBV fusion protein (gB) and entry of Epstein-Barr Virus (EBV) into B cells of the immune system. Three viral glycoproteins (gH, gL and gp42) form a well-defined complex with host receptor (HLA). We isolated the complex biochemically and studied its assembly by BLI biosensor and electron microscopy methods. Previous crystal structures revealed a hydrophobic pocket (HP) on the gp42 surface that when mutated disrupts fusion with B cells, but the critical binding ligand remained unknown. Our experiments show that the gp42 HP interacts with gHgL and that mutations of the predicted HP contact residues on gHgL are detrimental for fusion. Constraints imposed by the triggering complex architecture relative to its predicted membrane anchors highlight a close approach and potential deformation of both viral and host membranes affected by HLA receptor binding as a prerequisite to viral entry.

## Introduction

Epstein Barr Virus (EBV) or Human Herpesvirus 4 (HHV-4) is a *gammaherpesvirus* that is ubiquitous in humans and predominantly infects host epithelial and B cells, in which it establishes long-term latency. It is an oncogenic virus associated with a wide array of human tumors including epithelial cell tumors such as nasopharyngeal and gastric carcinomas, and lymphoid malignancies like Hodgkin and Burkitt lymphoma. Primary infection in children and young adults manifests itself as acute infectious mononucleosis. Subsequent reactivations of the virus are asymptomatic and managed effectively by the immune system in healthy adults. However, immunocompromised patients suffer from severe opportunistic disorders, such as post-transplant lymphoproliferative disease (PTLD), oral hairy leukoplakia and HIV/AIDS related malignancies [1].

Viral membrane fusion is a requisite step for infection for all lipid bilayer encased viruses, such as the herpesviruses, and requires one or several virus-encoded glycoproteins that orchestrate the merging of viral and host membranes in a step-wise manner [2]. This overall process leads to the release of viral capsid into the host cytoplasm initiating infection. The entry of EBV into B cells is complex and involves at least five different glycoproteins (EBV gp350/220, gH, gL, gp42 and gB) [3]–[6]. Of these five proteins, four (gH, gL, gB and gp42) are indispensable for membrane fusion with B cells and three (gH, gL and gB) are required for fusion with epithelial cells [7], [8]. gH, gL and gB form the core fusion machinery common to all herpesviruses. During B cell entry, EBV gp350/220 binds to complement receptor 2 (CR2/CD21) [9]–[11] concentrating virus to the B cell surface, but this interaction does not activate membrane fusion or virus entry. The gp42 protein forms stable, high affinity complexes with the gHgL complex [12], and also binds to human leukocyte antigen (HLA) class II [13] which acts as the triggering receptor for EBV entry into B cells [4].

The role of gHgL in herpesvirus entry has remained unclear, but most recently it has been suggested to act primarily as a regulator of gB activation rather than as a direct participant in driving membrane fusion [14]. gB is the most conserved glycoprotein in the herpesvirus family and it belongs to the class III viral fusion protein group, which includes the VSV G and baculovirus gp64 fusion proteins [15]. The putative post-fusion crystal structures of HSV and EBV gB have been solved [16], [17] and functional studies have highlighted its role as the likely fusion protein, with two critical fusion loops that are thought to interact directly with the host membrane [18]–[20]. Based on its structural similarity with the VSV G [21] protein, herpesvirus gB is thought to undergo a large conformational change that would drive virus-cell membrane fusion [4].

A key role of EBV gp42 is in determining the cell tropism of the virus. Binding of gHgL/gp42 complexes to HLA class II drives B cell infection, but gp42 binding to gHgL inhibits EBV entry into epithelial cells [4], [8], [22]. This inhibitory effect can be recapitulated by peptides containing ∼33 residues from the N-terminus of gp42 [23], which bind gHgL with similar high affinity as intact gp42. Epithelial cell entry is thought to require a direct interaction between gHgL and integrin receptors (αvβ5, αvβ6, and αvβ8 but not αvβ3), distinguishing at least two distinct modes of entry mediated by gHgL [24]–[26] associated with the two different physiological target cells of the virus.

EBV gp42 is a multifunctional type II glycoprotein, with an N-terminal domain of ∼100 amino acids and a C-terminal C-type lectin domain (CTLD). The N- and C- terminal domains engage gHgL and HLA, respectively, to mediate B cell membrane fusion and virus entry. We previously reported the structures of gp42 in the presence and absence of HLA class II [27], [28]. The complex structure revealed that HLA class II binds to the gp42 CTLD (residues 94–221) only through the HLA β-chain. The gp42 structures have shown that its N-terminal region (residues 33–93) is extended and mostly disordered. Gp42 binds gHgL through this flexible N terminal region with nanomolar affinity [12], [23] and the interaction has been mapped to gp42 residues 36–81 [29]. We also identified a hydrophobic pocket (HP) located at the gp42 CTLD canonical binding site [27], [30], which is important for its ability to trigger membrane fusion subsequent to HLA binding. The role of the hydrophobic pocket in the gp42 CTLD has remained elusive, but we have observed that it undergoes a small structural change after gp42 binding to HLA [27], [28], which could be important in activating membrane fusion. Mutations within this pocket inhibit fusion, but not binding to gHgL or HLA, highlighting its functional importance in B cell fusion [30]. Finally, the gp42 ectodomain is cleaved *in-vivo* after the N-terminal transmembrane domain (residues 9–29) and this cleavage is required for productive viral fusion [31].

EBV gHgL is a heterodimeric protein with a rod-shaped structure (100 Å long and 30–60 Å wide) having four domains arranged linearly one after the other [32]. gL is entirely contained in domain I (D-I), which also includes the N-terminal 65 residues of gH. Mutations that affect membrane fusion are found throughout the EBV gHgL molecule, but studies have identified clusters of important residues within the N- and C-terminal domains and the D-I/D-II interface [33]–[36]. Structural comparisons with HSV-2 gHgL and PRV gH structures also reveal conformational differences in the interdomain arrangements, which could have functional significance [37], [38]. In particular, the D-I/D-II interdomain arrangement of EBV gHgL differs from HSV-2 gHgL, giving rise to a large groove present only in the EBV gHgL structure [32], [37]. This D-I/D-II groove is adjacent to a KGD motif implicated in binding integrins for mediating EBV entry into epithelial cells. Mutation of the KGD loop also has effects on gp42 binding and B cell fusion [39].

Here we study the assembly and structure of the biochemically reconstituted gHgL/gp42/HLA triggering complex. We demonstrate that the two gp42 ligands, gHgL and HLA, bind essentially independently of each other, consistent with HLA receptor binding inducing limited conformational changes in the viral protein complex [28]. We determined the 29-Å resolution structure of the complex by negative-stain electron microscopy (EM), demonstrating that it can adopt open and closed conformations and revealing that the functionally important gp42 HP interacts with gHgL. We further demonstrate that mutations located at this novel gHgL-gp42 interface disrupt membrane fusion. The overall architecture of the B cell triggering complex suggests that it may participate in early stages of EBV-mediated B cell entry by bringing viral and target cell membranes into closer proximity, and by positioning key residues in gHgL to engage the gB fusion protein.

## Results

### *In vitro* assembly of gHgL/gp42/HLA-class II triggering complexes

**Figure 1:**
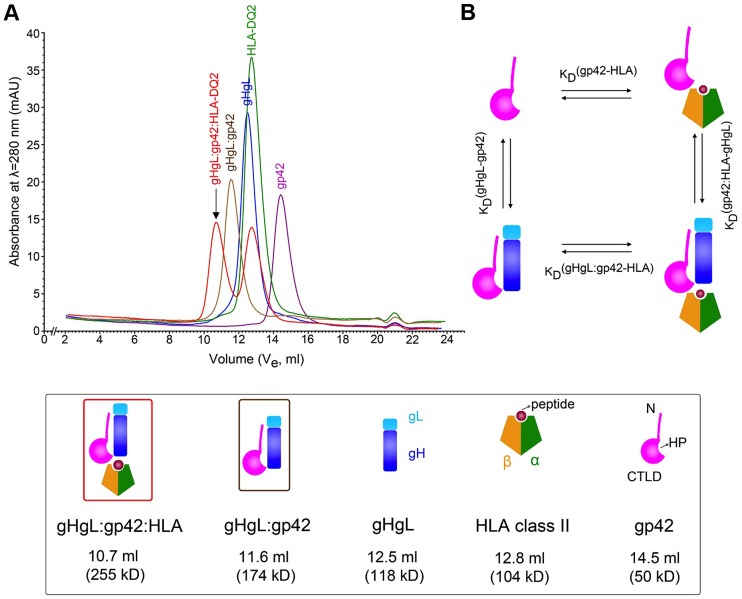
Biochemical assembly of the EBV B cell triggering complex. (A) *In-vitro* assembly of the EBV gHgL/gp42/HLA-DQ2 triggering complex (red, indicated by arrow) using size exclusion chromatography (S200). The triggering complex elutes at 10.7 ml (Ve, elution volume) with an estimated apparent MW of 255 kDa (also see Table 1). This complex is formed from EBV gHgL/gp42 complex (brown) mixed with excess HLA-DQ2 (green). Excess HLA-DQ2 can be seen as a second individual peak in the red trace. EBV gHgL/gp42 complex (brown) is formed quantitatively from 1∶1 molar mixture of gHgL (blue) and gp42 (purple). (B) Thermodynamic cycle linking the two pathways to the formation of the triggering complex. The horizontal (top and bottom) reactions represent the binding of HLA to either free gp42 or to gHgL/gp42 complexes, respectively. Similarly, the vertical (left and right) reactions represent the binding of gHgL to either gp42 or gp42/HLA complexes.

We previously demonstrated that EBV gHgL and gp42 form a tight 1∶1 complex [12] and that the holo-complex with a representative HLA molecule, HLA-DR1, can be prepared and isolated biochemically [40]. Here we expanded these studies to encompass another HLA isotype, HLA-DQ2, which also serves as an EBV B cell entry receptor [4], [41], [42]. The individual proteins, gHgL, gp42 and HLA-DQ2, were expressed and purified and their interactions initially characterized by gel filtration chromatography (Figure 1A). The purified proteins exhibited homogenous single peak profiles consistent with their approximate molecular weights (Table 1). Using a Superdex 200 gel filtration column, the gp42 protein eluted at volume of ∼14.5 ml corresponding to an estimated MW of 50 kDa, the HLA-DQ2 eluted at ∼12.8 ml (apparent MW ∼104 kDa) and the gHgL eluted at ∼12.5 ml (apparent MW ∼118 kDa) (Figure 1A). Addition of gp42 to gHgL resulted in the quantitative formation of a new, higher molecular weight peak eluting at ∼11.6 ml (MW ∼174 kDa), consistent with previous observations [12]. The addition of excess HLA-DQ2 to preformed gHgL/gp42 complex resulted in the formation of an even larger complex eluting at 10.7 ml with an estimated apparent MW of ∼255 kDa, consistent with HLA-DQ2 forming stable complexes similar to those observed with HLA-DR1 [40] (Figure 1A). The components of each peak were verified by SDS-PAGE (data not shown), further demonstrating the recruitment of all proteins into the larger ∼255 kDa complex peak. The elution volumes and estimated molecular weights of the proteins and complexes are collected in Table 1. Similar complexes could be assembled with HLA-DQ2 loaded with two different peptides in the HLA peptide-binding groove (both endogenous and exogenous, in the form of CLIP1 and α1 gliadin peptides respectively), consistent with a minimal impact of HLA-bound peptides on the gp42 interaction and EBV entry. We refer to the reconstituted gHgL/gp42/HLA-DQ2 complex as the “triggering complex” for B cell entry.

**Table 1:** Size exclusion chromatography elution volume (V_e_) and apparent MW for the gHgL, gp42 and HLA-DQ2 proteins and complexes.

Glycoprotein or glycoprotein complex	Number of glycoprotein components	Elution Volume (V_e_, ml)	Apparent MW^1^ (kDa)
gp42 w.t.	1	14.5	50
gp42 (C114S)	1	14.6	48
gp42 (I159C)	1	14.4	52
HLA-DQ2 (α1)	1	12.8	104
gHgL	2	12.5	118
gHgL/gp42 wt	3	11.6	174
gHgL/gp42 (I159C)	3	11.7	166
gHgL/gp42/HLA-DQ2 (α1)	4	10.7	255
gHgL/gp42/HLA-DQ2 (CLIP1)	4	10.8	245
gHgL/gp42(C114S)/HLA-DQ2 (CLIP1)	4	10.7	255
gHgL/gp42(I159C)/HLA-DQ2 (CLIP1)	4	10.7	255
gHgL/gp42(I159C)mPEG2K/HLA-DQ2 (CLIP1)	4	10.8	245
gHgL/gp42(I159C)mPEG10K/HLA-DQ2 (CLIP1)	4	9.9	360

1 as calculated by the following formula based on superdex 200 calibration (linearized, semi-log plot)
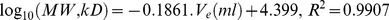

The specific peptide loaded into the HLA-DQ2 peptide binding site is indicated in parentheses (α1–α1 gliadin peptide; CLIP1 – class invariant chain derived peptide).

### Kinetic and thermodynamic binding constants governing triggering complex assembly

Studies of the gp42 structure alone and in complex with HLA-DR1 suggested the potential for conformational changes to be induced in the gp42 hydrophobic pocket (HP) after receptor binding [27], [28]. Such conformational changes could relay the information from HLA receptor binding to enable gHgL activation of gB-mediated membrane fusion. We therefore investigated quantitatively the assembly of the triggering complex, in order to establish if the energy of HLA receptor binding might be used to initiate conformational changes in gHgL/gp42.

The assembly of the gHgL/gp42/HLA complex can proceed from the isolated proteins along two pathways that form a closed thermodynamic cycle (Figure 1B). The binding of two ligands to gp42 is not necessarily independent and there are a number of mechanistic scenarios in which binding of gHgL and HLA to gp42 could be cooperative or competitive. If gp42 binding to gHgL is independent of HLA binding to gp42, then the interaction of gp42 or gHgL/gp42 complexes with HLA-DQ2 would have identical affinities (Figure 1B; top and bottom of the reaction cycle). Similarly, if the binding of these two gp42 ligands is independent, then gp42 binding to gHgL should be identical to the binding of gp42/HLA-DQ2 complexes to gHgL (Figure 1B; left and right of the reaction cycle). However, if the binding of HLA to gp42 induces, for example, conformational changes that are transmitted to gHgL, or if gHgL and HLA molecules exhibit any other form of coupling energetics upon binding to gp42, this would be reflected in changes in the binding affinities of bound vs. free gp42 with either ligand.

**Figure 2:**
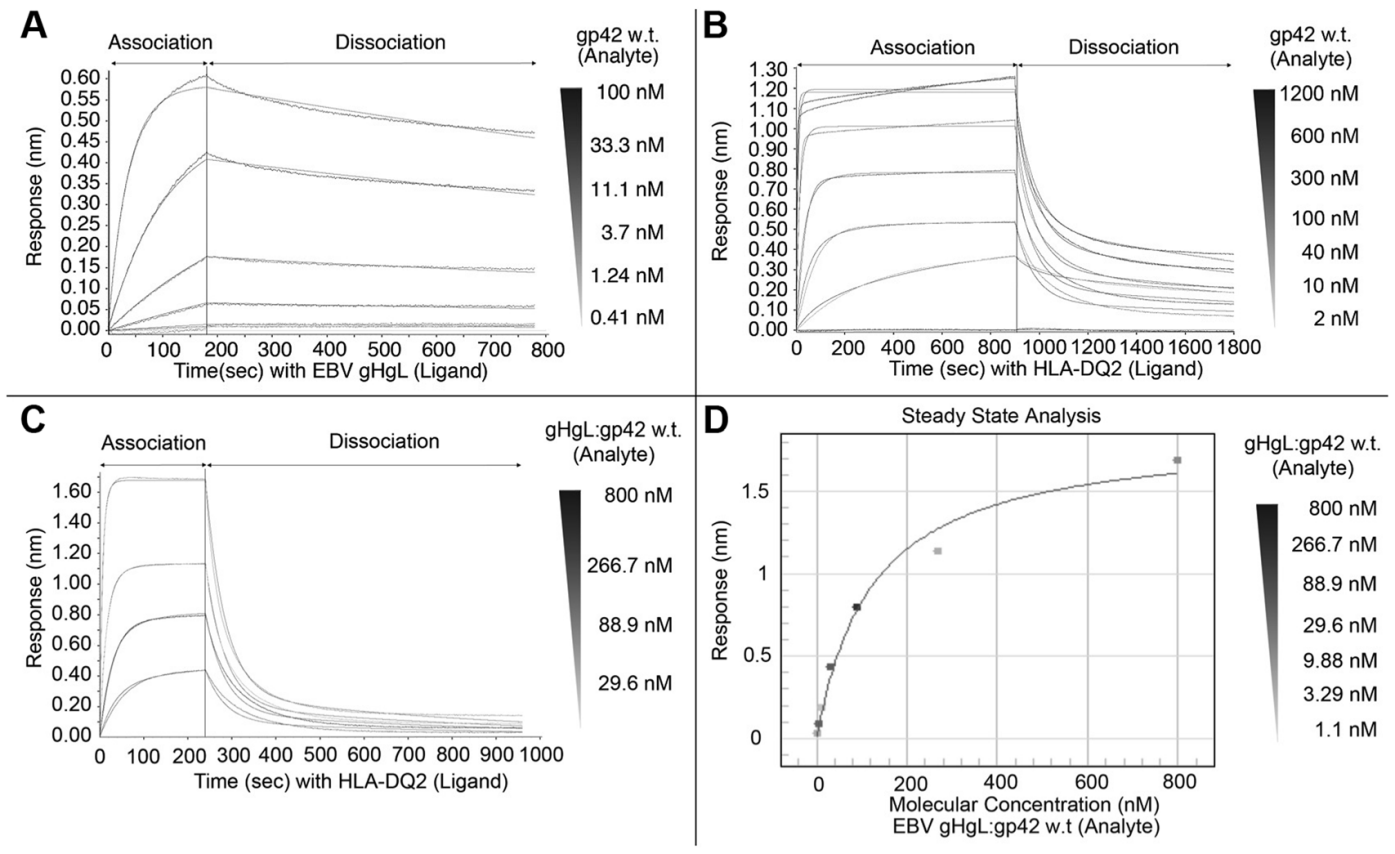
BioLayer interferometry (BLI) binding studies with wildtype gp42. Binding kinetics were measured with the Octet RED96 instrument (ForteBio, Pall Corporation) using wildtype (wt) gp42 protein. (A) Biotinylated EBV gHgL was immobilized on streptavidin (SA) biosensor tips and incubated over a range of concentrations (0.4–100 nM) of soluble wt gp42. (B) Biotinylated HLA-DQ2 (CLIP1) was immobilized on streptavidin biosensor tips and incubated over a range of concentrations (2–1200 nM) of soluble wt gp42. (C) Immobilized HLA-DQ2 (CLIP1) was incubated with increasing concentrations of preformed gHgL/gp42 complexes (30–800 nM). Data was fit globally to different binding schemes corresponding to a 1∶1 langmuir binding isotherm (A) and a 2∶1 heterogeneous ligand binding model (B and C). (D) Steady state analysis of the gHgL/gp42 binding to HLA-DQ2 shown in C for equilibrium K_D_ determination. Fitted kinetic and equilibrium binding constants are collected in Table 2.

To examine the binding rates and affinities associated with the formation of the gHgL/gp42/HLA complex, we used biolayer interferometry (BLI) binding methods using a ForteBio Octet RED96 biosensor instrument (Figure 2). In a first set of experiments, biotinylated gHgL was immobilized on Streptavidin (SA) biosensor tips and gp42 binding was studied by varying its concentration over the range of 0.4–100 nM (Figure 2A). Previous experiments using fluorescence polarization (FP) to measure the binding between gHgL and gp42-derived peptides have estimated the binding affinity to be on the order of 1–5 nM [23], [29]. The Octet binding data was fit globally with a 1∶1 interaction model, providing an overall affinity value (K_D_) of 1.4 nM for the EBV gHgL interaction with gp42 (Figure 2A and Table 2) and a t_1/2_ for dissociation of ∼30 minutes. Equilibrium analysis of the BLI data provide an independent measure of the K_D_ of ∼4 nM, consistent with the kinetic analysis. The biosensor binding data are in overall good agreement with the peptide FP K_D_ estimate, suggesting that the N-terminus of gp42 does provide the majority of the binding energy between gp42 and gHgL.

**Table 2:** Bio-layer Interferometry (BLI) Binding Kinetics with Biotinylated EBV gHgL and HLA-DQ2 with gp42 variants and gHgL/gp42 complex.

*Binding of gHgL to gp42*				
Analyte	Kinetic Analysis 1			Equilibrium Analysis
	k_on_, M^−1^s^−1^ (×10^5^)	k_off,_ s^−1^ (×10^−4^)	K_D_ (nM)	K_D_ (nM)
gp42 w.t.	2.75±0.01	3.88±0.02	1.41	4.1±0.93
gp42 C114S	4.09±0.04	5.29±0.04	1.29	6.3±3
gp42 I159C	3.81±0.02	2.95±0.02	0.77	13±2.8

*Binding of HLA-DQ2 to gp42 and gHgL/gp42*							
Analyte	Kinetic Analysis 2						Equilibrium Analysis
	k_on1_M^−1^s^−1^ (×10^5^)	k_off2,_ s^−1^ (×10^−4^)	k_on2_, M^−1^.s^−1^ (×10^5^)	k_off2_, s^−1^ (×10^−4^)	K_D_ (nM)	K_D2_ (nM)	K_D_ (nM)
gp42 w.t.	2.44±0.05	131±0.79	1.39±0.03	4.54±0.07	53.6	3.26	53±11
gp42 C114S	2.22±0.03	197±0.78	2.09±0.05	7.21±0.08	88.8	3.45	97±15
gp42 I159C	2.02±0.05	119±0.88	1.69±0.03	2.12±0.06	59.0	1.25	29±8
gHgL/gp42	1.92±0.03	227.8±1.17	1.06±0.05	14.48±0.24	118.9	13.64	120±22

1 1∶1 Global Fit.

2 2∶1 Heterogeneous Ligand Fit.

errors represent standard deviations (±S.D) of the fitted kinetic constants.

To facilitate the measurement of gp42 binding to HLA-DQ2 in the presence and absence of gHgL, biotinylated HLA-DQ2 loaded with the CLIP1 peptide was immobilized onto SA biosensor tips. Binding of the wt gp42 protein was investigated over the concentration range of 2–1200 nM (Figure 2B). The data were fit best using a 2∶1 heterogeneous ligand binding model, providing affinity values for the primary component (K_D1_) of 54 nM and a binding constant of 3.2 nM for a secondary minor component (K_D2_, Table 2). The 2∶1 heterogeneous ligand model does not represent two ligands binding simultaneously to a target protein, but is a model for 2 ligand species binding with different binding constants. This may arise from heterogeneity in the gp42 or the HLA proteins. Because gp42 has a tendency to aggregate, the heterogenous ligand kinetics could reflect the interaction of monomeric vs. aggregated gp42 with HLA-DQ2. The K_D1_ closely follows the overall independent K_D_ value of 53 nM obtained upon steady state analysis of the same data (Table 2), confirming the K_D_ obtained through kinetic analysis.

Complexes of gHgL/gp42 were freshly prepared and isolated by gel filtration chromatography for comparative binding studies with biotinylated HLA-DQ2 (Figure 2C). The concentration of the gHgL/gp42 complexes was varied over the range between 1–800 nM by serial dilution. Global curve fitting of the binding data with a variety of models was attempted and a 2∶1 heterogeneous ligand model gave the best fit (Figure 2C), yielding a K_D1_ value of 118 nM (Table 2). The overall equilibrium affinity could be independently estimated from the saturation of the binding curves, providing a K_D_ value of ∼120 nM for the formation of the ternary complexes, which is in good agreement with the kinetic K_D1_ (Figure 2D and Table 2). Attempts to quantitatively measure gHgL binding to preformed gp42/HLA complexes using the biosensor were confounded by the weaker affinity and faster dissociation rate of gp42/HLA compared to gp42/gHgL complexes.

Overall, these data indicate that the binding affinity of gHgL/gp42 with HLA-DQ2 is within 2-fold of gp42 alone, with a free energy difference of <0.5 kcal/mol, suggesting that there are no major energetic interactions coupling the binding of these two ligands to gp42. The results indicate that HLA receptor binding does not induce major conformational changes in the gHgL/gp42 complex that require receptor binding energy, and that gp42 binds its two ligands essentially independently. Previous studies have shown that the gp42 hydrophobic pocket undergoes a small change in structure upon HLA binding [27], [28], consistent with the conclusions of these binding studies.

### Electron Microscopic structure of the triggering complex

**Figure 3:**
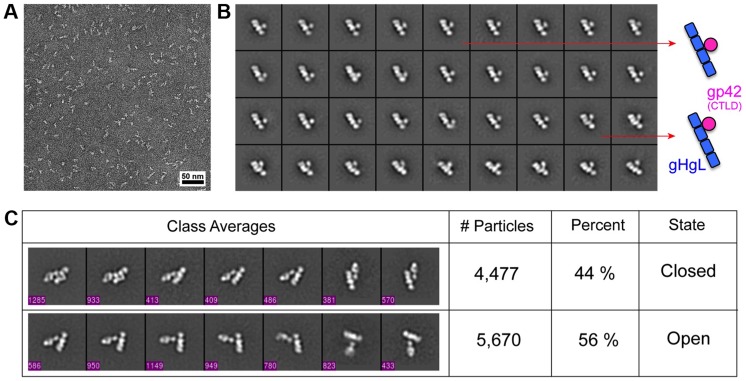
Negative-stain electron microscopic (EM) image classificaion of the EBV gHgL/gp42/HLA-DQ2 triggering complex. (A) A representative negative-stain EM image of gHgL/gp42/HLA-DQ2 sample. (B) Representative class averages of gHgL/gp42. Shown to the side is a schematic representation depicting the variability in the gp42 CTLD position along the length of gHgL before HLA-class II binding. (C) Representative class averages of gHgL/gp42/HLA-DQ2 complexes in open and closed conformations in ∼50∶50 proportion. The particle numbers included in the ternary complex classes are also indicated in each class average in (C).

Purified gHgL/gp42/HLA-DQ2 complexes were examined by negative-stain EM (Figure 3A). The images revealed that the sample was composed of two major types of particles: gHgL/gp42/HLA-DQ2 and gHgL/gp42 and only 5–10% of total particles represented intact gHgL/gp42/HLA-DQ2. The dissociation of HLA-DQ2 might result from the low sample concentration required for the negative-stain EM corresponding to ∼50 nM, which is close to the measured K_D_. To reconstruct the 3D structures of both gHgL complexes, homogeneous particles were selected by image classification and the 3D reconstructions were carried out using the random conical tilt (RCT) method [43] implemented in the SPIDER software [44].

Class averages revealed that the gHgL/gp42 complexes adopted different arrangements of the gp42 CTLD relative to gHgL (Figure 3B). The CTLD appears to be able to migrate along the length of the gHgL rod-like structure, while remaining in relatively close proximity to the gHgL surface. These observations are consistent with gp42 binding with high affinity through a flexible peptide segment located within its N-terminal 86 residues, followed by a short flexible linker separating the N-terminal domain and the CTLD [23], [27], [29]. The EM observations further indicate that the gp42 CTLD does not interact strongly with gHgL.

Analysis of the gHgL/gp42/HLA-DQ2 complexes shows that these exist in at least two states that are approximately equally populated (Figure 3C). Nearly 50% of the complexes adopt a “closed” conformation in which gHgL and HLA-DQ2 molecules appear to be more closely aligned, whereas the other 50% adopt a more heterogenous “open” state (Figure 3C). For the open state, the two arms of the ternary complex vary in orientation with angles of ∼40°–115° relative to each other.

**Figure 4:**
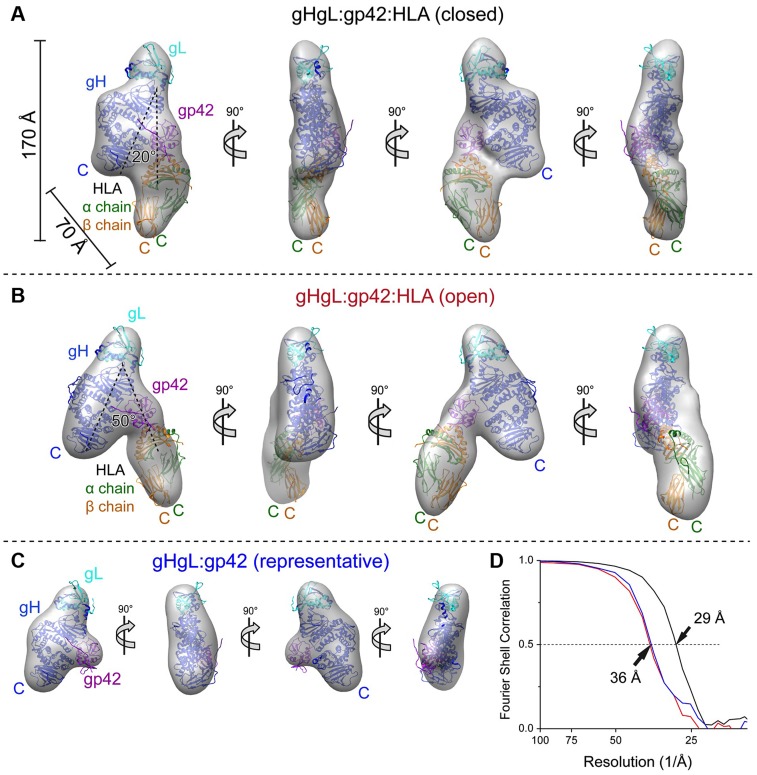
RCT 3D reconstructions of the EBV gHgL complexes. EM structure of the intact triggering complex in the closed (A) and a representative open (B) conformation fit with the known crystal structures of gHgL, gp42 and HLA class II. A unique solution to the fitting was obtained either with EBV gHgL (3PHF) and gp42/HLA-DR1 complex (1KG0) (depicted here), or EBV gHgL (3PHF) and gp42 (3FD4) and HLA-DQ2 (1S9V). EBV gH and gL are shown in blue and cyan, respectively. EBV gp42 represented in hotpink, and HLA-DR1 shown in green (α-chain) and orange (β-chain). (C) EM reconstruction and fitting of the component crystal structures of a representative conformational state of the gHgL/gp42 complex. (D) Fourier shell correlations (FSC) of the RCT 3D reconstructions from 1,219, 604, and 657 65°-tilted particle images for the closed complex (black), the open complex (red), and the gHgL/gp42 subcomplex (blue), respectively. The resolution of the 3D reconstruction is estimated at FSC = 0.5.

Representative 3D EM maps of the ternary and gHgL/gp42 complexes were obtained from particle classes with estimated resolutions of 29 Å (“closed” state), 36 Å (“open” state), and 36 Å (gHgL/gp42 complex) (Figure 4). The successful reconstruction of the ternary complex indicates that the gHgL/gp42/HLA-DQ2 complex adopts a relatively homogeneous architecture, particularly in the closed conformational state. Although the “open” gHgL/gp42/HLA-DQ2 and gHgL/gp42 complexes are more variable, representative 3D reconstructions for these complexes were also generated (Figure 4B,C). The EM maps were used for fitting the known component crystal structures of gHgL (PDB ID: 3PHF) and the gp42/HLA-DR1 complex (PDB ID: 1KG0). The structures fit unambiguously in the maps (Figure 4A–C), positioning gp42 near to gHgL, with HLA-DQ2 projecting away from the viral protein complex in both the open and closed conformations. In the closed conformation, gHgL and gp42/HLA-DQ2 are in an approximately parallel arrangement with HLA-DQ2 in proximity to gH D-IV (Figure 4A). In the “open” conformation, gp42 is rotated by about 30°, leading to gp42/HLA-DQ2 tilting towards a more perpendicular orientation relative to gHgL (Figure 4B). The gHgL/gp42 complex lacking HLA-DQ2 is similar to its counterpart in the intact complex (Figure 4C).

**Figure 5:**
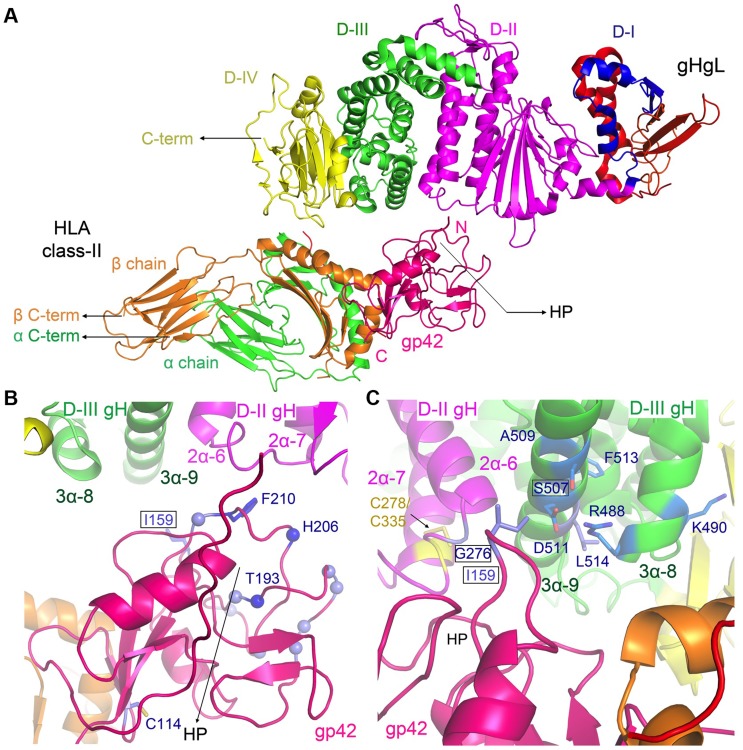
The pseudoatomic model of the triggering complex places the gp42 HP in contact with gH. (A) Pseudo-atomic model of the gHgL/gp42/HLA-DQ2 complex obtained from the EM envelope fitting. The individual domains of each protein are marked and the hydrophobic pocket (HP) in gp42 is highlighted with an arrow. The C-termini of gH and HLA chains are similarly marked and lie on one side of the complex at ∼70 Å of each other. (B) The gp42 HP interaction site with gHgL. Two sides of the gp42 HP interact with gH between D-II and D-III, including a loop between helices 2α-6 and 2α-7 from D-II and helix 3α-9 from D-III. Residues that have been previously mutated in the gp42 HP [30] are indicated, H206 and T193 that had linker insertions as Cα spheres (dark blue) and F210 as sticks (dark blue) within the hydrophobic pocket (light blue Cα spheres) as defined in [27], [28], which disrupt membrane fusion activity. I159 is also shown as sticks (light blue). The gp42 HP faces away from the observer in this orientation. The gp42 interaction with the HLA-class II β-chain (orange) brings gH and HLA into close proximity. Residue C114 which is the only unpaired cysteine in wt gp42 is shown as sticks (light blue) (C) Close-up view of gp42 residue I159 (blue), located in the gp42 158 loop [28], at the junction of the HLA and gH contact sites. The locations of gH mutant residues that were tested are indicated, including G276 (cyan) in the 2α-6/2α-7 loop (D-II) and D511 and S507 in the 3α- helix 9 (D-III). The view is rotated 180° along vertical axis and then rotated 45° counter-clockwise with the horizontal axis compared to the orientation shown in (A) and (B). Images were rendered using MacPyMol [55].

The pseudo-atomic EM models place the gp42 hydrophobic pocket (HP) at the interface between D-II and D-III in gHgL (Figure 5A). The interface involves two sides of the gp42 HP, which interact with gH residues in a loop between D-II helices (2α-6 and 2α-7; residues K275–E282) and a D-III helix (3α-9 helix; residues Q503–E520) (Figures 5B, 5C). Although specific contacts cannot be conclusively identified at this resolution, gp42 residues that have been previously shown to be important for triggering membrane fusion are located at the interface. Linker insertion mutants at gp42 residues 193, 206 and 210, as well as the point mutation of F210A, reduce membrane fusion but do not affect binding to gHgL or HLA receptor [30], and these are located close to the gH interface (Figure 5B). The structure suggests that the well-defined architecture of the triggering complex is at least in part due to interactions between the gp42 CTLD and gHgL, which is mediated through the gp42 HP. Mutations in the gp42 HP may therefore disrupt membrane fusion activity by specifically affecting the gp42 CTLD interaction with gHgL.

The composite EM model also positions the C-termini of gH and the HLA-class II ectodomains to one side of the triggering complex (Figure 5A), within ∼70 Å of each other in the closed complex. This model suggests that the formation of the triggering complex could affect the approach of the viral and cellular membranes prior to membrane fusion. The gH protein transmembrane (TM) domain lies within ∼6 residues from the observed C-terminus of the gH ectodomain, placing the viral membrane in close proximity to the observed structure. Superposition of HLA-DQ2 onto the HLA-DR1 in our model predicts that ∼9 residues link the observed C-termini of the HLA α and β chains to their respective TM domains and the target cell membrane (Figure 5A). Interestingly, the gp42 ectodomain must be cleaved from its N-terminal TM domain to be active in membrane fusion [31], suggesting a potential geometric constraint in this assembly. No structural information is available on the location of the N-terminus of gp42 that is proximal to the membrane, as this is a flexible region of ∼60 amino acids (residues 33–94) that are involved in high affinity gH binding. Although the structures incorporated into our EM model lack the gH and HLA TM and cytoplasmic domains, there is no evidence that these regions would affect the overall ectodomain conformations.

### A mutation in the gp42 “158 loop” specifically disrupts membrane fusion

Residues in a loop (the ‘158’ loop) at the junction of the gp42 HP and the HLA binding site have been implicated in mediating conformational changes to the gp42 HP after receptor binding [28]. Previous mutagenesis studies demonstrated that mutations in the 158 loop could affect both HLA binding and membrane fusion, but mutations at the tip of this loop were not tested to see if these showed specific defects in membrane fusion but not HLA binding [27], [30]. In the EM model of the complex, residue I159, which resides at the tip of the 158 loop, is oriented to interact with the gH D-III helices near residues 492 and 507 (Figure 5B,C). We mutated I159 to cysteine to test its effects on membrane fusion and to also allow additional labeling of gp42 at this site. Since wt gp42 has an unpaired cysteine at residue 114, this was also mutated to serine to avoid secondary labeling outside of the 159 position. Both the C114S single mutant and C114S/I159C double mutant (referred to as I159C) were expressed and purified to homogeneity for membrane fusion studies. The ability of the mutants to stimulate fusion with B cells was measured in a cell-cell fusion assay in which CHO cells transfected with gB, gHgL and a T7 luciferase reporter construct are mixed with Daudi B cells expressing T7 RNA polymerase. The addition of soluble gp42 to these cells triggers fusion, which is quantified by measuring luciferase activity [7].

**Figure 6:**
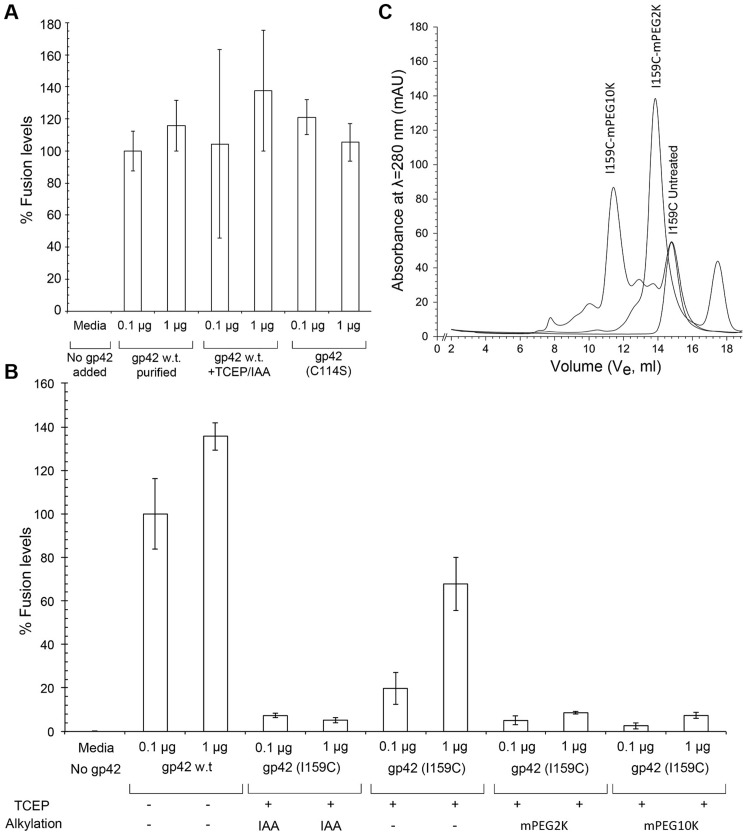
Modifications of gp42 residue I159 disrupt membrane fusion with B cells. In panels (A) and (B), CHO cells were transfected with gB, gHgL, and a T7-Luciferase reporter and either 0.1 µg or 1.0 µg soluble purified gp42 or gp42 mutant was added 24 hours post transfection along with T7 expressing Daudi B cells. The negative control (F12 media) was similarly transfected and mixed with Daudi cells but no soluble gp42 protein was added. (A) The wildtype gp42 residue C114 is not important for membrane fusion activity. Treatment of wt gp42 with reducing (TCEP) and alkylating (IAA) agents, to irreversibly block the C114 –SH group, does not affect gp42 membrane fusion activity. Mutation of C114 to serine also does not affect membrane fusion activity, it also has no unpaired cysteines due to the mutation and hence is not treated with reducing agent or alkylating agent. (B) Mutation and chemical modifications of gp42 residue 159 block membrane fusion activity. Fusion assay results of wt gp42, I159C (reduced with TCEP), I159C (reduced and alkylated with IAA) and I159C site-specifically pegylated with maleimidePEG 2000 and 10000 MW_avg_ (denoted as mPEG2K and mPEG10K). (C) Gel filtration traces of gp42 I159C along with gp42 I159C modified with mPEG2K or mPEG10K. The shift in elution volume (V_e_) with increasing molecular weights of PEGylation is evident, allowing purification of the appropriately modified gp42 protein for fusion assays and complex formation with gHgL and HLA for EM imaging. The peaks for the purified fraction of the gp42 mutant protein are denoted as I159C-Untreated, I159C-mPEG2K and I159C-mPEG10K from right to left respectively. Abbreviations: TCEP is Tris(2-carboxyethyl)phosphine, hydrochloride; IAA is Iodoacetamide and, mPEG stands for maleimide-polyethylene glycol and mPEG2K or mPEG10K denotes this chemical with a MW_avg_ 2000 or 10,000 Da.

First, we established that the free cysteine at residue 114 in wt gp42 is not important for its function. The wt gp42 protein was subjected to reduction with Tris(2-carboxyethyl) phosphine, hydrochloride (TCEP) and alkylation with iodoacetamide (IAA) to potentially block the free thiol and this did not affect fusion activity (Figure 6A). The gp42 C114S mutant also showed fusion levels comparable to wild type gp42 (Figure 6A), further indicating that the cysteine is not a functionally important residue.

We then tested the effects of the I159C mutation in the C114S background. The I159C mutant, treated with either TCEP alone, to reduce the 159C thiol, or with TCEP & IAA, to reduce and alkylate the introduced cysteine, showed a significant defect in membrane fusion activity. The reduced and alkylated I159C protein activity dropped to below 10% of wild type levels, (Figure 6B). However, when the I159C mutant was only reduced with TCEP and not alkylated, it retained some residual membrane fusion activity, suggesting that covalent modification of the cysteine at residue 159 by IAA was in part responsible for reducing the mutant protein function (Figure 6B).

In order to demonstrate that specific modification of residue 159 was affecting gp42 function, we modified the I159C position with larger, more easily identifiable substituents by reacting the mutant protein with maleimide-PEG 2,000 (mPEG2K) and maleimide-PEG10,000 (mPEG10K). Both pegylated I159C proteins could be purified to homogeneity by gel filtration chromatography (Figure 6C). The modification could be verified by observable shifts in apparent MW in gel filtration chromatography for both the mPEG2K and mPEG10K modified proteins (Figure 6C) and by SDS-PAGE for the mPEG10K modified protein. Fusion assays were conducted with the gel filtration purified, PEG-modified I159C mutants (Figure 6B). The pegylated I159C mutants all gave levels of membrane fusion of 5–10%, similar to the alkylated gp42 I159C protein, indicating that PEGylation disrupted fusion activity similarly to the alkylated gp42 I159C protein (Figure 6B).

**Figure 7:**
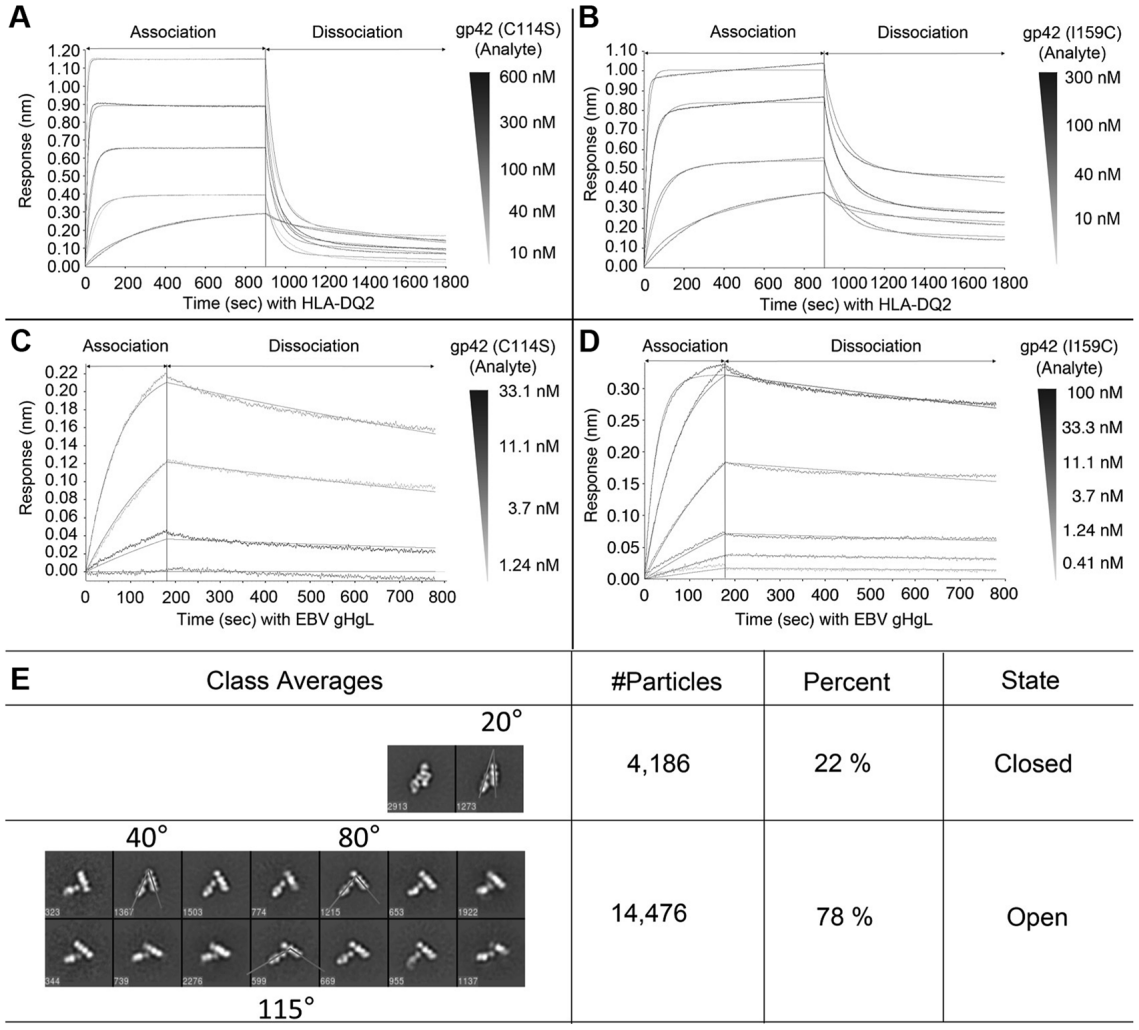
Mutation of gp42 I159 does not affect binding affinity or kinetics with gHgL or HLA-DQ2. Kinetic binding experiments were conducted similarly to Figure 2 on the OctetRED96. For panels A and B site-specifically biotinylated HLA-DQ2 (CLIP1) was immobilized on the streptavidin (SA) biosensor tip surfaces. Binding kinetics of HLA-DQ2 (ligand) with the single mutant gp42 C114S (A) or the gp42 I159C double mutant (B) are depicted. Global curve fitting with a 2∶1 heterogeneous ligand model closely matched the experimental data. The calculated binding curves are shown overlaid on the data from the experiment. For panels C and D, biotinylated EBV gHgL was immobilized on the streptavidin (SA) biosensor surfaces. Binding kinetics of EBV gHgL with the single mutant gp42 C114S (C) or I159C double mutant (D) are depicted. Overlay curves show the global fitting results using a 1∶1 Langmuir binding model. Kinetic and thermodynamic binding constants are similar to binding gp42 wildtype (Figure 2 and Table 2). (E) Representative class averages of gHgL/gp42-PEG2K/HLA-DQ2 complexes. The gp42 I159C mutant was labeled with PEG-2K maleimide and used to form complexes with gHgL and HLA-DQ2, which were by purified gel filtration chromatography. Representative angles between the two arms of the complexes formed by gHgL and gp42/HLA are indicated as well as the number of particles included in each class.

We investigated the binding of the I159 mutant to both gHgL and HLA-DQ2 to rule out possible effects of the mutation on binding to either of these gp42 ligands. Octet biosensor binding data, obtained with both the single C114S mutant and the I159C/C114S double mutant, show nearly identical binding of the mutants to both gHgL and HLA-DQ2 proteins as compared to wt gp42 (Figure 7 and Table 2). The membrane fusion experiments demonstrate that mutation of the tip of the gp42 158 loop results in similar defects in membrane fusion as other gp42 HP mutations. However, in contrast to studies of previous HP mutants, here we demonstrate quantitatively that the I159 mutation has no significant effects on either the binding affinities or kinetics for gHgL or HLA interactions, but nonetheless membrane fusion is inhibited. These data further indicate that the gp42 HP interactions with gHgL are important in fusion activation, but do not contribute significantly to the high affinity tethering of gp42 to gHgL.

We biochemically prepared triggering complexes with the IAA-treated gp42 I159C mutant (gHgL/I159C/HLA-DQ2) to investigate its structure by EM, but did not observe any major differences in the structures. The IAA-treated 159C complexes also showed an essentially identical distribution between open (47%) and closed (53%) complexes. We also prepared triggering complexes with pegylated I159C mutant (gHgL/I159C-mPEG-2K/HLA-DQ2). Stable PEGylated gHgL/gp42/HLA-DQ2 complexes could be isolated by gel filtration chromatography (Table 1) and these resulting complexes were examined by negative-stain EM (Figure 7E). Although the PEGylated gp42 complexes appear to be structurally similar to the wt triggering complex, the distribution of particles in the open and closed conformations shifts to ∼78% open and 22% closed from the ∼50∶50 distribution observed with wt gp42, suggesting that disrupting the closed conformational state of the triggering complex could be functionally important.

### Mutations in gHgL at the gp42 HP interface also disrupt membrane fusion activity

**Figure 8:**
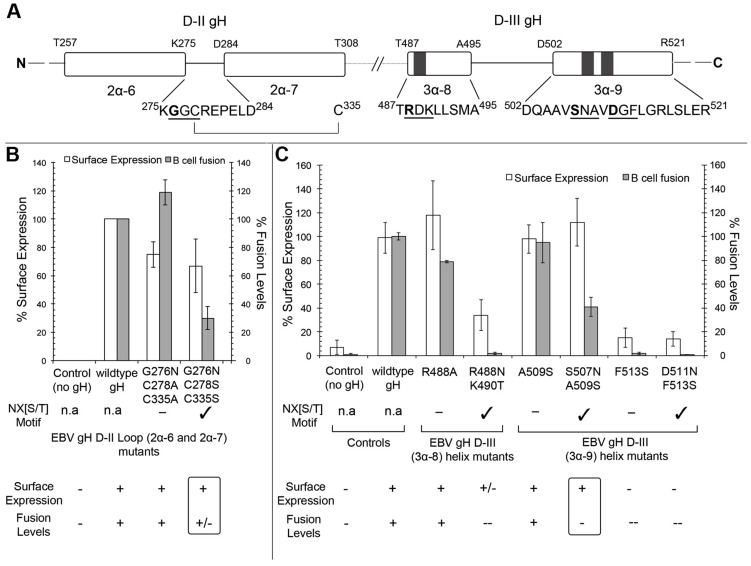
Mutations in gH at the interface with the gp42 HP affect membrane fusion. Cell surface expression and fusion assay results with B cells with gH mutants in the R488, S507 and D511 regions. Cell surface expression was measured using the anti-gHgL monoclonal antibody E1D1. (A) The gH D-II and D-III sequence observed proximal to the gp42 HP from our EM model has been highlighted to show the sequence and secondary structure indicating the mutual positions of the mutations in gH that would validate the EM model. The residue mutated to Asn (N) that gets potentially glycosylated is shown in bold text, the NX[S/T] motif is underlined. Cystine bridge between C278–C335 is highlighted; EBV gH D-II and D-III mutants to validate the position of residues in gH close to HP as revealed in our EM model are studied in the following panels (B) and (C); (B) gH G276N in the background of the disulfide bond mutant C278A/C335A does not have the glycosylation motif NX[S/T] and exhibits near wildtype fusion levels with B cells. By contrast, the G276N/C278S that introduces the glycosylation motif at 276 reduces fusion levels to lower than 40%. (C) The R488A mutation does not have a significant effect on gHgL expression or B cell fusion, while the R488N/K90T mutation reduces expression and membrane fusion. The D511N/F513S mutant and its control (F513S) show drastically reduced surface expression and B cell fusion. Both gH507N/A509S and the control gH A509S are expressed near wildtype levels and the gH S507N glycosylation mutant shows a reduction in membrane fusion activity. The mutants where the surface expression is as good as the gp42 wildtype but the fusion levels are down (below ∼40% or less) are highlighted in the rounded rectangular box. Mutants that result in potential glycosylation due to the introduced residue change are shown with a check mark.

Based on the EM model, we generated mutations in gH at the predicted interface with gp42 to test their effects on membrane fusion (Figure 5C). Given the low resolution of the structure and the associated difficulty in accurately predicting gH-gp42 contacts, we designed mutations that introduce an NX[S/T] consensus motif predicted to generate novel N-linked glycosylated sites at the interface. The mutants studied include a gH DII mutant G276N/C278S/C335S with a nonglycosylated G276N/C278A/C335A control (Figure 8A,B). Since C278 and C335 form a disulfide bond in wt gH, these two residues were mutated together, to avoid leaving a reactive free thiol at this site. Additional interface mutants in DIII included D511N/F513S, S507N/A509S, R488N/K490T, with corresponding controls that would not introduce the NX[S/T] glycosylation motif (Figure 8A,C). The DIII mutants D511N/F513S and F513S mutants were not expressed well and showed no activity in fusion (Figure 8C). The R488N/K490T mutant exhibited a significant reduction in B cell fusion function, but with a concomitant decrease in cell surface expression (Figure 8C), whereas the control R488A point mutation expressed similar to wildtype and had only a slight effect on B cell fusion. The S507N/A509S and the A509S mutations both expressed similarly to wildtype. While the A509S mutation alone had no effect on membrane fusion, the S507N/A509S double mutant showed a reduction of ∼50% in membrane fusion with B cells (Figure 8C), consistent with its position near the gp42 HP in the triggering complex structure.

The introduction of a novel N-linked glycosylation site at gH residue G276 (Figures 5C, 8B) also reduced membrane fusion activity. The mutation of G276N/C278A/C335A does not introduce a NX[S/T] motif for glycosylation at position 276 and despite reduced cell surface expression, B cell fusion activity is slightly enhanced compared to wt gH (Figure 8B). Introduction of the glycosylation site in the G276N/C278S/C335S mutant resulted in a strong reduction in B cell membrane fusion, consistent with a disruption of interactions with the gp42 HP predicted by the EM model (Figure 8B). These data indicate that the 276 and 507 regions of gH are important in B cell membrane fusion, although significant disruption of fusion requires the introduction of larger perturbations than the selected point mutations tested here. Overall, the data support the conclusion that both sides of the predicted gp42-gHgL interface are functionally important for B cell membrane fusion activity.

## Discussion

**Figure 9:**
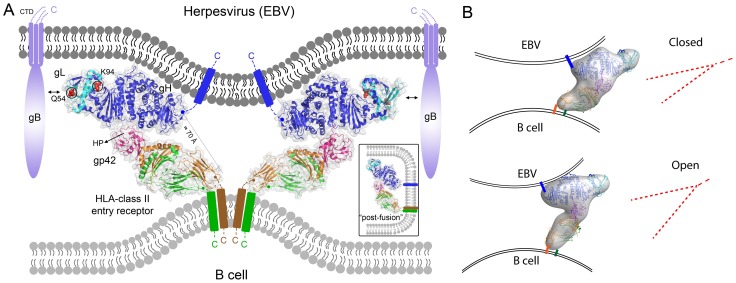
EBV B cell fusion model based on the gHgL/gp42/HLA (“triggering complex”) structure. (A) Model of the EBV B cell triggering complex in the context of viral and cellular lipid bilayers, with gHgL (blue/cyan), gp42 (hot pink) and HLA class II (green/orange). Locations of the transmembrane (TM) domains of gH and HLA- are indicated schematically with rectangles spanning the viral and cellular bilayers on one side of the complex and a separation of ∼70 Å. The C-termini of gH and HLA class II are also indicated. Although gp42 is a type II transmembrane protein, the N-terminal TM domain must be cleaved for it to be active in fusion and the resulting location in the triggering complex of the gp42 mature N-terminus is not known. The ∼170 Å long complex suggests a skewed orientation of the proteins relative to the two bilayers that could bridge viral-cellular membranes, potentially distorting and/or otherwise preparing the site for subsequent membrane fusion. This model places gL residues (Q54 and K94) involved in gB activation on the opposite side of the gH and HLA membrane anchors. The external location of gB implied by this model may indicate an initial peripheral activation of gB followed by its movement to a more central position to mediate membrane fusion. The gp42 HP, which is critical for fusion activation and located at the gH D-II/D-III junction, is highlighted to show its location with respect to the triggering complex. A potential postfusion arrangement of the triggering complex is show in the inset to the lower right. (B) Schematics of the V/Y shape of the open and closed triggering complexes highlighting their similarity to the structures observed in cryo-ET studies of HSV-1 entry described in [48].

It has been proposed that the herpesvirus gHgL protein might play various roles in membrane fusion, potentially participating directly in the mechanics of membrane fusion [45] or alternatively by acting purely as a regulator of gB activation and leaving the fusion process entirely to the fusion protein gB [4], [32] The process by which receptor binding by gHgL or gHgL/gp42 leads to gB activation may involve gHgL conformational changes, but it has remained largely unclear. Here we studied the assembly and structure of the EBV B cell triggering complex, demonstrating that there is no significant energetic coupling between the binding of the two gp42 ligands, gHgL and HLA, suggesting that receptor binding energy is not required to initiate large conformational changes internally in the complex. We also determined the negative-stain EM structure of the triggering complex, observing that it adopts a well-defined closed state and a variable open conformation. Docking of the crystal structures of the component gHgL, gp42 and HLA proteins into the EM map provided a pseudo-atomic model of the complex. In addition to indicating that the B cell triggering complex forms well defined arrangements of the proteins, the EM models reveal three additional unanticipated observations. First, the gp42 CTLD forms direct contacts with gHgL at the junctions of D-II/D-III (Figures 4A, 5A). Second, the gp42 HP, a region that is critical for fusion activation, is positioned at the interface with gHgL (Figure 5B,C). Third, the C-terminal ends of gHgL and HLA-DQ2 are oriented to one side of this complex, suggesting that the assembly of the triggering complex may bring the viral and cellular membranes into proximity (Figures 4A, 5A, 9). We demonstrated that mutations of residues in both gp42 and gH at the gp42 HP-gH interface reduce membrane fusion with B cells (Figures 6B, 8B, 8C), further validating the EM model.

Our model of the B cell triggering complex answers several important questions that arose from previous structural and functional studies [4]. The gp42 HP lies above the gp42 HLA class II binding site and is required for B cell fusion. Mutations in the pocket abrogate fusion [30], but not HLA or gHgL binding. Importantly we demonstrated here that mutation of gp42 residue I159, implicated by the EM structure as interacting with gHgL, also blocked membrane fusion, but did not alter the binding kinetics or affinity of gp42 for gHgL or HLA-DQ2. It had previously been postulated that gB or gHgL might interact with the gp42 HP [28], but our EM model and functional studies clarify that gH is the HP ligand.

We demonstrated that IAA- and PEG2K-modified gp42 159C proteins are both defective in activating membrane fusion, but EM images of the triggering complexes appear generally similar to the wt complexes. Interestingly, the PEG2K modified gp42 mutant did reveal a change in the percentage of complexes in the closed conformation, suggesting that alterations in the stability of this conformation could be responsible for the membrane fusion defect. However, given that the IAA-treated I159C complexes appear similar to wt, it may not be possible to discern small but critically important conformational changes in the low-resolution EM complexes that are responsible for the loss of function. It is also possible that changes in the dynamic stability of the complexes governed by the gp42-HP:gHgL interaction may not be evident in the distribution of closed and open states in the IAA-treated mutant, but this could potentially explain a reduction in fusion efficiency.

The EM structure of the closed triggering complex reveals that the C-termini of membrane anchored gH and HLA-DQ2 would be positioned within 70 Å of each other and located to one side of the complex at the bilayer-bilayer interface during membrane fusion (Figure 9). C-terminal residues of gH [35] and the membrane anchor [46] itself have been implicated as important in fusion, although contradictory results have suggested that soluble gHgL could either activate or inhibit membrane fusion comparing HSV and EBV [46], [47]. Studies using cryo-electron tomography (cryo-ET) of HSV-1 fusion in two model systems, adherent cells and synaptosomes, show distinct V/Y shaped structures (∼15 nm) bridging the viral and cellular membrane prior to membrane fusion [48], which were interpreted as representing gHgL and/or gB proteins. The vitreous ice frozen structures reveal dimpling of the lipid bilayer, as well as smaller structures near the site of membrane pinching and these V/Y shaped structures. This study also indicated that only a few glycoprotein complexes might be involved in organizing the viral and cellular membranes near the site of fusion [48]. The gHgL/gp42/HLA pseudo-atomic model that we have determined shares this overall V/Y shape. The predicted intermembrane distances of ∼10 nm for the closed gHgL/gp42/HLA complex is also similar to the bridging structures observed in the HSV studies, suggesting that these may represent a common structural feature formed at initial, prefusion stages of herpesvirus entry. Our mutagenesis studies of the gp42 HP and gH contact sites further indicate that this bridging structure plays a critical role in promoting membrane fusion events.

The orientation of the EBV triggering complex relative to the viral and cellular membranes is unknown. However, comparison to the V/Y structures observed in the HSV-1 cryo-ET study suggests that the C-termini of the gH and HLA ectodomains might be oriented towards the central site of closest approach of the viral and cellular bilayers (Figure 9). The open and closed conformations of the triggering complex observed in the EM studies suggest that assembly of the complex, and specifically formation of the closed conformation, could play a role in drawing the viral and cellular membranes into closer proximity. The model of the triggering complex positions gHgL D-I on the opposite side of the structure from the TM domains anchored in the viral and cellular membranes and binding HLA receptor could potentially position D-I closer to the viral membrane surface (Figure 9). Two gL residues (Q54 and K94) located at the tip of D-I are critical for the specificity of gB activation [33]. This placement of D-I away from the gH and HLA TM domains may enable the recruitment of gB to the triggering complex through the oriented gHgL D-I site. Once gB is activated, it could then potentially move from an initially peripheral location to a more central region of the virus∶cell interface to directly mediate fusion between the two membrane bilayers.

Based on these observations, we propose a model for EBV entry into B cells, in which the triggering complex plays two potential roles, by bringing the viral and cell membranes into closer proximity, potentially through an open-closed conformational transition, and by orienting gHgL to enable the activation of gB-mediated fusion (Figure 9). The well-defined architecture of the gHgL/gp42/HLA assembly could induce dimpling or distortions of the viral and cellular membrane bilayers to accommodate the complex. The short linker of gH to the viral membrane may explain why gH D-IV residues have been shown to be important in membrane fusion [35], [36], as they could potentially play a role in deforming the viral bilayer. The orientation of gHgL complexes relative to the two bilayers may enable gB recruitment and activation. Finally, the merger of the viral and host membranes may relieve membrane distortions induced by gHgL/gp42/HLA complexes, allowing the transmembrane domains to relocate into the same final bilayer structure (inset, Figure 9). By contrast, studies with epithelial cells have indicated that soluble integrin receptor binding to gHgL may be sufficient to trigger membrane fusion [26], suggesting potential differences in the energetics or process for activating gB. Further comparative studies of EBV epithelial cell triggering complexes and energetics are required to understand how infection of these two cell types is orchestrated by EBV fusion glycoproteins.

## Materials and Methods

### Expression and purification of EBV gHgL, gp42 variants and HLA-DQ2

Detailed instructions for the expression and purification of EBV gHgL and gp42 have been previously published [12]. Baculovirus vector expression system (BD Biosciences) was used for making the soluble constructs of EBV gH, gL and gp42. EBV gH residues 18–679 and gL residues 24–137 were fused to a gp64 leader sequence for secretion with the resultant addition of three N-terminal residues AMT for gH and AMD for gL each under the expression control of the p10 and polyhedron promoters respectively. The gHgL protein expressed in this study had no purification tags. Gp42 residues 33–223 were cloned into the pBacGus-3 vector, with the gp64 signal sequence and N-terminal 6-His and S tags as previously described [12]. ESF921 media (Expression systems) and HyQ media (Hyclone) were used for growing Sf9 or High Five insect cells (Invitrogen) and SF+ cells (Protein Science, Meriden, CT) respectively. Sf9 or High Five cells were maintained at 1 million cells/ml and SF+ at 1.5 million cells/ml every 48 hrs at 27°C incubator shaking at 135 rpm. Sf9 cells in monolayer were used for serial passage of baculovirus stock production in T-flasks with complete TNM-FH media (BD Biosciences). SF+ cells were used for gHgL expression and High Five cells for gp42 expression with corresponding 2% v/v P3 (fourth generation) baculovirus stock to cells at 1.5–1.8 million/ml at 27°C shaking at 135 rpm. Typically ∼4 L of SF+ cells expressing gHgL and 1–2 L of High Five cells expressing gp42 were processed. The expressed secreted protein was harvested three days (72 hrs) post-infection. The secreted proteins were isolated by affinity chromatography. E1D1 antibody coupled to an Ultralink hydrazide resin column (Pierce, Thermo Scientific) was used for purifying untagged gHgL, and metal-affinity resin (Talon, Clontech or Ni-NTA resin, Qiagen) used for purifying six His-tagged gp42 wildtype and the gp42 mutants (C114S single and I159C double mutant). EBV gHgL was eluted with 0.1 M glycine pH 2.5 and neutralized immediately with 1 M Tris pH 8 stock and sodium chloride to final concentration 150 mM. Alternatively, EBV gHgL was also eluted with gentle Ag/Ab elution buffer pH 6.6 (Pierce, Thermo Scientific). Gp42 was eluted with 20 mM Tris, 150 mM NaCl, 300 mM Imidazole, pH 7.4. Both gHgL and gp42 proteins were buffer exchanged to 20 mM Tris, 150 mM NaCl, pH 7.4. E1D1 hybridoma was generously provided by L. Hutt-Fletcher (Louisiana State University Health Sciences Center, Shreveport, LA). The hybridoma was expanded to get soluble E1D1 protein supernatant from the National Cell Culture Center (NCCC, Minneapolis, MN). The gHgL and gp42 proteins were polished using a Superdex 200 10/300 GL column with 20 mM Tris, 150 mM NaCl, pH 7.4 as the final buffer.

The construct details for HLA-DQ2 have been previously described [49]. Briefly, our present study uses the A1*0501/B1*0201 HLA-DQ2 allele with a Fos-Jun leucine zipper pair that replaced the transmembrane regions and stabilize the αβ dimer, along with deamidated α1 gliadin peptide (QLQPFPQPELPY) or CLIP1 (PVSKMRMATPLLMQA) peptide covalently linked to the N-terminal end of the β-chain by a thrombin cleavable,15-aa linker. The C-terminal leucine zipper also could be cleaved post-expression using 3C protease and this was done for the protein used in EM analysis. The C-terminus of the β-chain following the zipper includes the eight amino acid FLAG tag sequence (DYKDDDDK) used as the purification tag, followed by a BirA enzyme site for site-directed biotinylation. This site was enzymatically biotinylated in vitro with Biotin-protein Ligase (Avidity LLC, Aurora, CO) to enable kinetic studies with streptavidin (SA) biosensor tips. Stably transfected S2 insect cells (from *Drosophila melanogaster*) were used for the production of aforementioned FLAG-tagged HLA-DQ2 protein with CLIP1 (endogenous) or α1 gliadin (exogenous) peptides. S2 cells were maintained between 4–10 million cells/ml using complete Schneider's media (Invitrogen Gibco 21720) supplemented with 5% FBS (Atlanta Biologicals S12450, after heat-inactivation) and 2 mM final glutamine concentration (Gibco 25030, 200 mM stock). Cells were amplified by gradually diluting the FBS out into the final Baculogold Max-XP serum-free insect cell media (BD Pharmingen 551411) with added 2 mM glutamine. Cells were grown by shaking at 120 rpm in a 27°C incubator and induced for HLA-DQ2 expression with final concentration of 1 mM copper sulfate at a cell density of 6 million/ml. Media supernatants were passed through ANTI-FLAG M2 affinity gel resin (A2220, Sigma-Aldrich, St.Louis, MO) and eluted by competing with free FLAG (DYKDDDDK) peptide (F3290, Sigma-Aldrich, St.Louis, MO or RP10586-1, Genscript USA Inc. Piscataway, NJ). The eluted HLA-DQ2 protein was concentrated and passed through Superdex 200 (GE Healthcare Life Sciences) as the final purification step.

### Bio-layer Interferometry (BLI) kinetics

Label-free binding interaction analyses between gp42, gp42 C114S single mutant or gp42 I159C double mutant and soluble HLA-DQ2 (CLIP1) in the presence or absence of gHgL were performed on the Octet RED96 (ForteBio, Pall corporation). All interactions studies were performed with the Streptavidin (SA) dip-and-read biosensors (ForteBio). Soluble HLA-DQ2 (CLIP1) or EBV gHgL was biotinylated for the different assays. HLA-DQ2 was site-specifically labeled whereas EBV gHgL was labeled randomly through primary amine linkage with biotin at 1∶3 molar ratio using the EZ-link NHS-PEG4 biotin kit (Thermo Scientific). Comparison of acquired K_D_ estimates to previously obtained measurements from fluorescence polarization (FP) for gHgL binding to gp42 [29] suggest that the random biotinylation does not affect the binding interaction.

### Kinetic and equilibrium K_D_ analysis methodology

Kinetic analysis by global fitting of model parameters (*k_on_*, *k_off_*, *K_D_*, *k_obs_* representing on rate, off rate, dissociation constant and observed rate respectively of complex formation between immobilized ligand and mobile analyte) across a range of analyte concentrations simultaneously were performed using the Octet Data Analysis software package version 7.0.1.3 (ForteBio, Pall Corporation). The 1∶1 global fit was modeled by the differential equation 
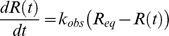
 giving values for *k_obs_*, *k_on_* from the association phase of the sensorgram and *k_off_* from the dissociation phase using binding data from a collective set of different analyte concentrations fit globally. Additionally, for true 1∶1 binding 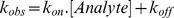. Further, 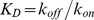 and equilibrium response or 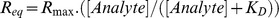 where *R*_max_ is the maximal response achievable by saturating the active binding site of the immobilized ligands with the mobile analyte. The 2∶1 heterogeneous ligand model with two independent binding events to two different ligand states was modeled by 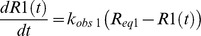 and 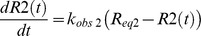. Additionally, 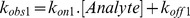 and 
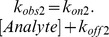
 with 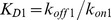 and 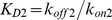. Total response, *R* = *R*1+*R*2. Equilibrium analysis was performed with the same data sets and modeled by the equation taking the average response at steady state or 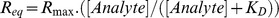 corresponding to a rectangular hyperbola.

### Modification of gp42 I159C in hydrophobic pocket (HP) with maleimide PEG 2000 and 10000

Purified gp42 I159C was treated with 4 mM TCEP pH 7.0 briefly and incubated on ice for 10 minutes to reduce to the cysteine sulfhydrl (–SH). This treated protein was mixed in 1∶100 w/w ratio with maleimidePEG 2000 or 10000 MW (Nanocs Inc.) and incubated at room temperature for one hour. The reaction was stopped by adding 50 mM iodoacetamide (IAA) in PBS pH 7.4 (final) to block any remaining reduced cysteine residues. Quantitative formation of new higher molecular weight species could be detected for gp42 modified on Superdex 200 elution profile (Figure 6C and Table 2) and on SDS-PAGE (data not shown). The PEGylated gp42 protein was used in complex formation with gHgL and HLA-DQ2 for EM studies and also in virus-free B cell fusion assays.

### Virus-free B cell fusion assay to test gp42 HP mutants and gH mutants

B cell fusion assays were performed as previously described [7]. Briefly, effector CHO-K1 cells were transfected with plasmids expressing gB, gHgL and the T7 luciferase reporter gene. Twenty-four hours post transfection, the cells were detached, counted and mixed 1∶1 with target cells expressing T7 RNA polymerase (Daudi 29 B cells), along with soluble wt gp42, gp42 C114S or gp42 I159C proteins as indicated, in a 24-well plate in Ham's F-12 medium with 10% heat-inactivated FBS. Twenty-four hours later, the cells were washed once with PBS and lysed with 100 µL of passive lysis buffer (Promega). Luciferase activity was quantified by transferring 20 µL of lysed cells to a 96-well plate and adding 100 µL of luciferase assay reagent (Promega) and luminescence was measured on a Perkin-Elmer Victor plate reader.

### Negative-stain three-dimensional EM reconstruction of the triggering complex

For negative-stain EM, 2 µL sample was applied to a glow-discharged grid coated with carbon film. After 30-second incubation, the sample was blotted with filter paper and stained with 0.8% uranyl formate. EM micrographs were recorded on a TIETZ F415MP 16-megapixel CCD camera at 68,027× calibrated magnification in an FEI Tecnai F20 electron microscope operated at 200 kV. The micrographs were saved by 2× binning, yielding a pixel size of 4.41 Å. The image acquisition was performed with the assistance of Leginon automation software [50], [51].

In total, 143 pairs of random conical tilt (RCT) images were collected for the sample of gHgL/gp42/HLA-DQ2 with the grids tilted at two angles successively (65° and 0°) for each specimen area of interest. 148,701 pairs of particles from tilt pairs were picked using *ApTiltPicker.py* in Appion [52]. To avoid bias in particle picking, all possible particles were picked for the following image classification. The defocus values of 0°-tilted and 65°-tilted micrographs were calculated by CTFFIND and CTFTILT programs [53], respectively. The phase-flipping was performed on particle images before classification and 3D reconstructions. The 0°-tilted particles were classified using the Correspondence Analysis method in SPIDER [44]. 3D RCT maps were reconstructed from 65°-tilted particles and iteratively refined with SPIDER by refinement of the center of 65°-tilted particles. Representative 3D RCT maps of the intact gHgL/gp42/HLA-DQ2 complex in the “closed” state, that in the “open” state, and the gHgL/gp42 subcomplex were reconstructed at 29 Å, 36 Å, and 36 Å resolution from 1,219, 604, and 657 particle images, respectively. These maps were used for fitting by known crystal structures of gHgL (PDB ID: 3PHF) and gp42/HLA-DR1 complex (PDB ID: 1KG0) using UCSF Chimera [54].

For the comparison of the percentage of the “closed” and the “open” states, the samples of gHgL/gp42/HLA-DQ2, IAA-treated gHgL/I159C/HLA-DQ2, and gHgL/I159C-mPEG-2K/HLA-DQ2 were negatively stained using the same condition. The particles from 50 micrographs of each sample were classified using the aforementioned method. The class averages corresponding to the “closed” and the “open” states were isolated by visual inspection.
